# A Supervised Video Hashing Method Based on a Deep 3D Convolutional Neural Network for Large-Scale Video Retrieval

**DOI:** 10.3390/s21093094

**Published:** 2021-04-29

**Authors:** Hanqing Chen, Chunyan Hu, Feifei Lee, Chaowei Lin, Wei Yao, Lu Chen, Qiu Chen

**Affiliations:** 1 Shanghai Engineering Research Center of Assistive Devices, School of Medical Instrument and Food Engineering, University of Shanghai for Science and Technology, Shanghai 200093, China; hqchen0503@163.com (H.C.); cwlin2019@gmail.com (C.L.); weiyao0721@163.com (W.Y.); lchenshijuesh@163.com (L.C.); 2 School of Optical-Electrical and Computer Engineering, University of Shanghai for Science and Technology, Shanghai 200093, China; hhuchy@163.com; 3 Major of Electrical Engineering and Electronics, Graduate School of Engineering, Kogakuin University, Tokyo 163-8677, Japan

**Keywords:** video retrieval, 3D CNN, supervised hashing, triplet loss

## Abstract

Recently, with the popularization of camera tools such as mobile phones and the rise of various short video platforms, a lot of videos are being uploaded to the Internet at all times, for which a video retrieval system with fast retrieval speed and high precision is very necessary. Therefore, content-based video retrieval (CBVR) has aroused the interest of many researchers. A typical CBVR system mainly contains the following two essential parts: video feature extraction and similarity comparison. Feature extraction of video is very challenging, previous video retrieval methods are mostly based on extracting features from single video frames, while resulting the loss of temporal information in the videos. Hashing methods are extensively used in multimedia information retrieval due to its retrieval efficiency, but most of them are currently only applied to image retrieval. In order to solve these problems in video retrieval, we build an end-to-end framework called deep supervised video hashing (DSVH), which employs a 3D convolutional neural network (CNN) to obtain spatial-temporal features of videos, then train a set of hash functions by supervised hashing to transfer the video features into binary space and get the compact binary codes of videos. Finally, we use triplet loss for network training. We conduct a lot of experiments on three public video datasets UCF-101, JHMDB and HMDB-51, and the results show that the proposed method has advantages over many state-of-the-art video retrieval methods. Compared with the DVH method, the mAP value of UCF-101 dataset is improved by 9.3%, and the minimum improvement on JHMDB dataset is also increased by 0.3%. At the same time, we also demonstrate the stability of the algorithm in the HMDB-51 dataset.

## 1. Introduction

In the past several years, video information has been widely used because of its richer content and it is easier to understand compared with other media. Due to the rise of various short video platforms and video sharing websites, a large amount of video information is uploaded to the Internet every day, which is widely shared through various social media and news platforms. People can also use various editing software to edit the video while browsing, such as inserting icons, changing the brightness, resizing, clipping video, and so on, which also increases the amount of video data. This not only leads to copyright infringement, but also makes video retrieval difficult, that is, people can hardly find the best matching videos. Usually, the results retrieved by computer need to be manually selected to find the most appropriate ones, which will increase the workload of users and affect the user experience.

As a result, efficient video retrieval algorithms have become nowadays an important component in the applications of copy detection [1], video recommendations [2], video retrieval [3] and copyright protection [4]. There are two main components in a typical content-based video retrieval (CBVR) system, one is feature extraction and another is similarity comparison. Traditional video feature extraction methods mostly based on individual video frames and hand-crafted features, like local binary patterns (LBP) [5], color histogram [6], or key-point descriptors (like SIFT [7]). For the past few years, CNN has been widely used for the ability of learning rich image representation, and widely used in classification task [8], scene recognition [9,10], object detection [11,12], face recognition [13], and image retrieval [14,15,16], etc. Not surprisingly, CNN is also used to solve video retrieval problems. However, video retrieval methods using 2D CNN often ignore the spatial-temporal connection between video frames. In order to prove the spatial-temporal consistency, a temporal network [17] is used to embed temporal constraints into the network structure for video retrieval. A temporal Hough voting scheme [18] is introduced to rank the retrieved database videos and estimate the segments that match the query. A method named learning to align and match videos (LAMV) [19] is used for aligning the videos temporally. A video similarity learning network named ViSiL [20] is proposed by first computing frame-to-frame similarity and then video-to-video similarity which avoids feature aggregation before the similarity calculation between videos. A method combining CNN to extract frame features and a recurrent neural network (RNN) to retain the temporal information is also proposed by [21], but RNN is hard to train due to the excessive number of parameters needed.

Compared with other kinds of content-based information retrieval, the difficulty of video retrieval lies in the fact that video information has more features that need a large amount of storage space and computing consumption. Benefiting from the XOR operation in binary space, hashing methods have advantages in retrieval efficiency and memory cost, but but previous hashing methods [22,23,24,25] are mostly adopted for image retrieval. Several video hashing methods [1,26] focus on getting better video feature representation instead of learning hashing functions. Many of the latest video hashing methods [27,28] are based on CNN + RNN, and a common problem of those methods is that too many parameters make it hard to train. From this point of view, what we need to solve at present is the problem of video feature extraction and retrieval efficiency in video retrieval.

To handle these problems, we propose in this research a novel method called deep supervised video hashing (DSVH) for large-scale video retrieval. First, we apply a pre-trained 3D CNN model to extract the temporal and spatial features in videos. Then, by using supervised hashing, the hash functions are trained, and features extracted by 3D convolution are mapped to binary space. Finally, by calculating the similarity of query video and dataset video in low dimensional space, the retrieval efficiency and accuracy can be improved greatly. The contributions of the proposed method are listed below:

(1) We design an end-to-end framework for fast video retrieval using the idea of a Deep Supervised Video Hashing. By learning a set of hash functions to transfer video features extract by 3D CNN to a binary space.
(2) We choose a fixed number of frames for each video to represent the characteristics of the entire video, which can greatly reduce the computation.
(3) We apply the idea of transfer learning and use a 3D CNN model with residual links pre-trained on large-scale video dataset to obtain the spatial-temporal features in videos.
(4) We conduct a great quantity of experiments over three datasets to demonstrate that the proposed method outperforms many state-of-the-art methods.

The following sections of the paper are arranged as follows: We begin with introductions of some related video retrieval works in Section 2. We introduce our proposed method in details in Section 3. Subsequently, in Section 4 we describe comprehensive experimental results of three datasets to verify the superiority of our method. At last, in Section 5 we summarize the article and our conclusions are presented.

## 2. Related Works

### 2.1. D Convolutional Neural Network

Convolutional neural networks have been used in many fields of computer vision due to their powerful feature processing capabilities, including video retrieval. Siamese convolutional neural network (SCNN) [29] is proposed to process video information, which contains two standard CNN networks for extracting video frame features. Wang et al. [30] propose a method that uses CNN to extract video frame features, and then obtains the video features by sparse coding (SC). In addition, Kordopatis-Zilos et al. [31] use a pre-trained CNN model to get the features of video frames, and then apply metric learning to video retrieval. All of these methods mentioned above have a common drawback is that they ignore the temporal relationship between frames, which leads to insufficient video feature extraction.

### 2.2. D Convolutional Neural Network

**Figure 1:**
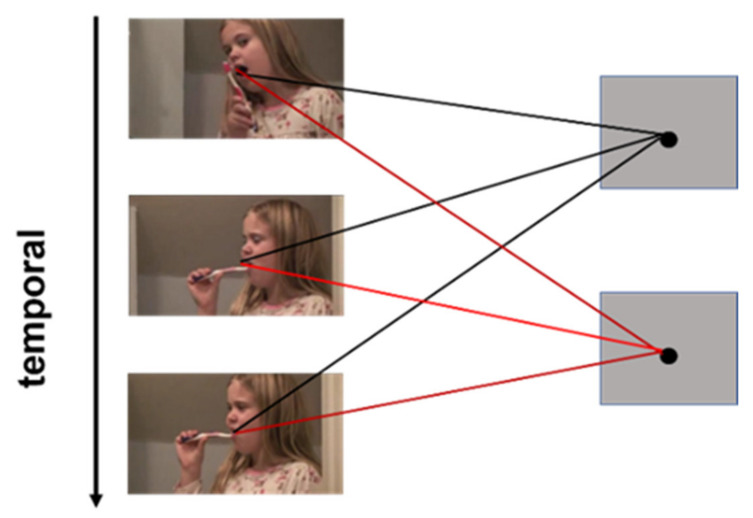
3D convolution.

In previous video retrieval methods using CNN for feature extraction, operations are usually carried out on single video frames. In this way, a significant drawback in processing video information is that the relationship between frames is ignored which certainly affects the precision of retrieval. Therefore, by performing 3D convolutions we expect to get spatial-temporal features of videos directly. 3D CNN [32] is first proposed for human action recognition, and then widely used for medical image processing [33] and video information processing [34] due to its spatial and temporal feature extraction ability. Some famous network models have been proposed, such as convolutional 3D [35], pseudo 3D [36], inflating 3D [37] and R (2 + 1) D [38]. As we can see from Figure 1, a 3D convolution operation is performed on the temporal and spatial dimensions of video frames arranged in chronological order and the feature map obtained is usually related to multiple video frames before and after, which can capture well the motion information and retain the features of the videos.

### 2.3. Hashing

Hashing is widely used for a variety of multimedia retrieval tasks with the purpose of transform the information from higher dimensional to lower dimensional, and it has attracted much interest in multimedia retrieval due to its search efficiency and memory saving features. According to whether label information is needed during the training step, we divide the existing hash methods into supervised and unsupervised hashing. Supervised hashing learns compact representations with the help of labels. Well known supervised hashing methods include: supervised hashing with kernels (KSH) [39] by minimizing the inner product of hash codes and minimizing loss hashing (MLH) [40] based on structural SVMs with latent variables and an effective online learning algorithm, etc. On the contrary, in unsupervised hashing methods, the label information is not necessary for learning hash function. Locality sensitive hashing (LSH) [41] is a typical unsupervised hashing method, which can ensure that the closer to the object, the greater the probability of collision. In addition, other unsupervised methods like iterative quantization (ITQ) [42] are also widely used. Various unsupervised hashing methods [43,44] are used in the field of image retrieval. Some unsupervised hashing methods are also used in video retrieval. Self-supervised video hashing (SSVH) [45] proposed an unsupervised video hashing framework for temporal nature of videos and learning to hash. Unsupervised deep video hashing (UDVH) [46] utilizes feature clustering and a specific rotation to balance the variance of each dimension.

### 2.4. Video Retrieval

With the development of multimedia information technology, people often need to retrieve videos from the Internet, and video retrieval technology has become a hot topic. Common types of video retrieval include text-based queries [47], audio-based queries [48], and video-based queries [49]. In this paper we focus on retrieving videos through video queries, that is, for a given video, finding similar videos in a database.

The two most important parts of video retrieval are feature extraction and similarity comparison. In recent years, many deep learning-based approaches have been proposed. Kumar et al. [50] propose a video retrieval method with feature extraction combining CNN and RNN. In [49], a neighborhood preserving hashing approach is used for video retrieval with a neighborhood attention mechanism. Furthermore, a central similarity quantization method is employed to mine the central similarity of features [51]. A similarity-preserving deep temporal hashing method is proposed by Shen et al. [27] through CNN + RNN for feature extraction, and a deep metric learning objective named l_2 All_loss based on the improved triplet loss to preserve the similarity within the class and the difference between the classes. In [28], frame-level features are passed to a bidirectional LSTM for temporal information, then a supervised hashing method is employed to get the binary codes of videos. However, most of these methods have too many parameters to be difficult in model training.

## 3. Proposed Approach

**Figure 2:**
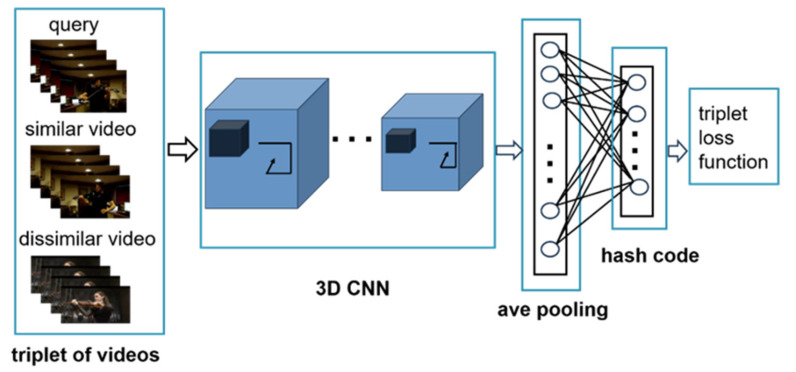
The proposed framework of video retrieval.

Figure 2 illustrates the proposed framework. The method we proposed is composed of three main components. The first step is to choose representative frames from the short video. The second component is to use a pre-trained 3D CNN to extract video features. The third is to fine-tune the network with the hash layer to get the hash function and then retrieval videos similar to the query.

### 3.1. Feature Extraction

#### 3.1.1. Frames Selection

**Figure 3:**
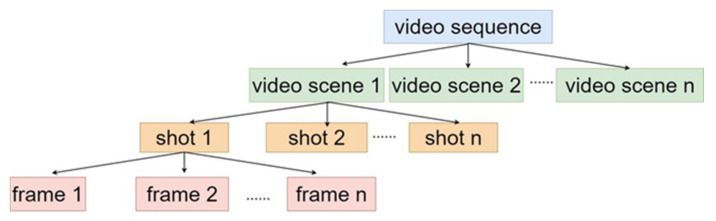
Typical structure of a video.

**Figure 4:**
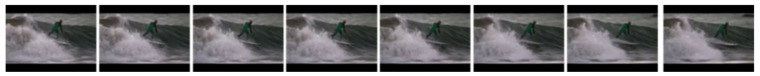
A continuous video frame sample in the UCF-101 dataset.

The first step of processing video data is to extract video frames. A video is composed of several video scenes, a video scene is composed of various shots, and a shot is composed of many video frames. As we can see from Figure 3, video frame is the basic unit of a video, even a few seconds long shot video may contain a huge number of video frames. For example, the usual video frame rate is 30 frames per second (FPS), which means that even a one-second video clip contains 30 video frames, but with the development of camera and storage technology, there are more and more videos with high FPS. Figure 4 shows a sample of video frames in UCF-101 dataset. We can see that and there is often only a little difference between adjacent video frames.

**Figure 5:**
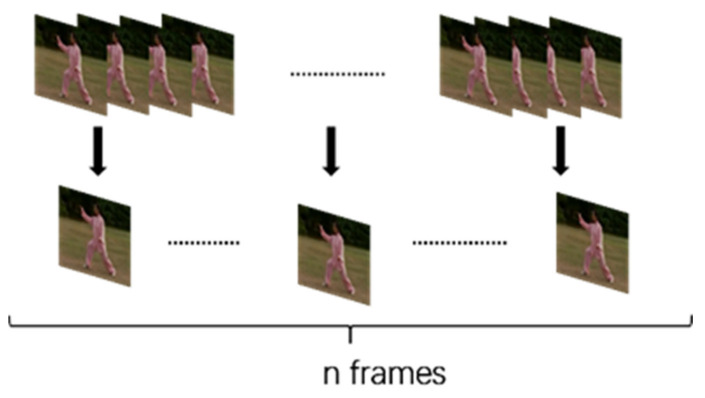
Selection of representative frames.

If we carry out feature extraction on all frames, the amount of computation is too large, and it is not necessary. Therefore, to reduce the computational amount and increase the retrieval efficiency, some representative video frames will be selected for feature extraction by certain strategies defined using Equation (1):

(1) $$f\left(i\right)=i\frac{t*FPS}{n}\left(i=1,2,\ldots ,n\right)$$

where t  represents the video length in seconds and FPS the frame rate, n is the number of frames selected to represent the video. The schematic diagram of representative frame selection is shown in Figure 5.

#### 3.1.2. Feature Extraction

**Figure 6:**
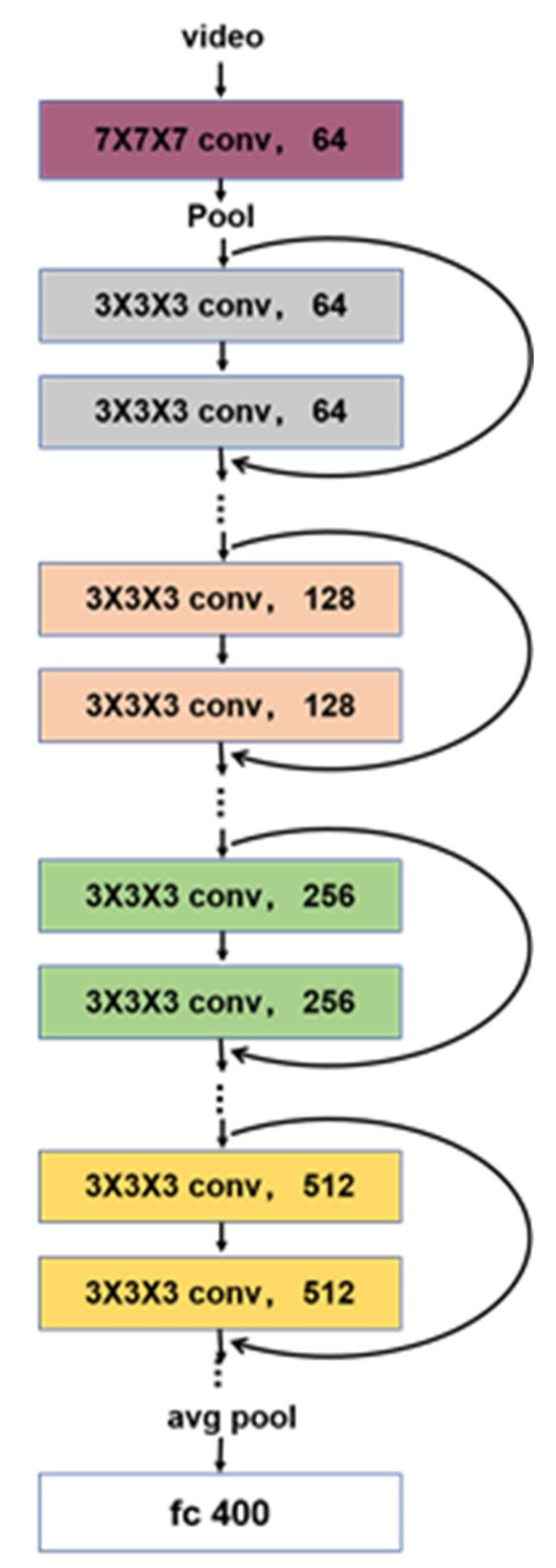
3D ResNet structure.

The CNNs pre-trained on ImageNet are used in various computer vision tasks and achieved great success. The application of CNN models pre-trained on large datasets that uses the idea of transfer learning greatly saves workload, saves time, and reduces the problems caused by insufficient training data. Inspired by the huge success of the pre-trained model, we adopt a pretrained 3D CNN model [52] for feature extraction, which structure and parameters of the network are shown in Figure 6 and Table 1. By applying residual modules [53] to 3D CNNs, we except to improve the retrieval accuracy. Then we fine-tune the model by adding a hash layer with a fully connected structure on the target dataset.

### 3.2. Hash Layer

**Figure 7:**
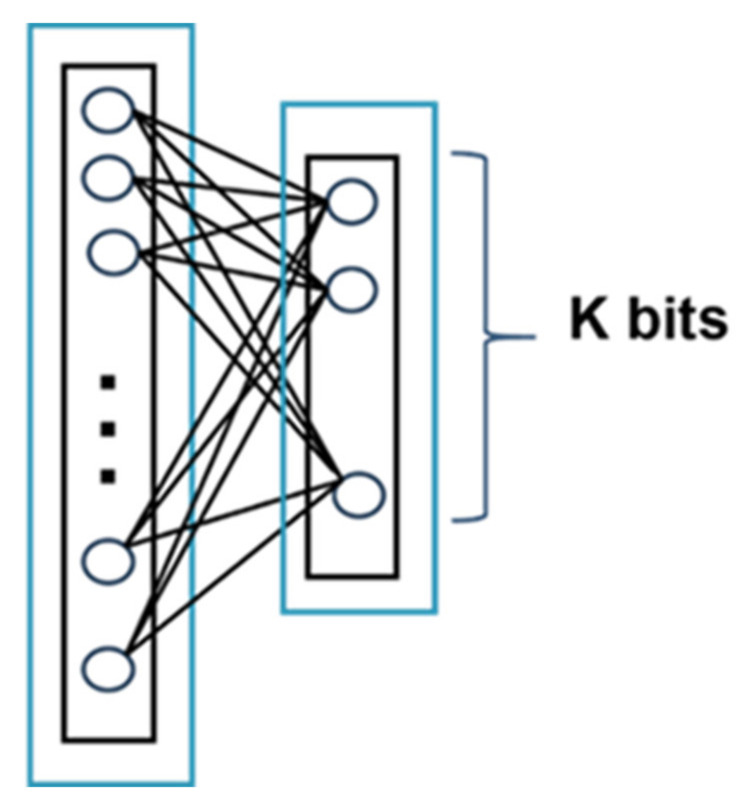
Hash layer structure.

After obtaining video features from the network with stacked convolutional layers, we need to map features extracted by 3D CNN to Hamming space for quick retrieval. We build a hash layer with fully connected structure as show in Figure 7 to map the feature extracted by convolution into binary space and get the representation of video use a k-bit vector. In the training step, in order to limit the features value extracted by 3D CNN to [–1, 1], we use the tanh activation function in the hash layer.

In the test retrieval step, in order to use hamming distance to measure similarity between videos, we need binarize the hash code of videos, so we define Equation (2) to generate the hash code. If the output value of the *j*-th bit is greater than or equal to 0, its corresponding hash code is 1; otherwise, it is −1:

(2) $$H^{j}=\left\{\begin{array}{l} 1,\;\;\;\;out^{j}\geq 0\\ -1,\;\;\;\;otherwise \end{array}\right.$$

### 3.3. Loss Function

In our proposed algorithm, we use the triplet ranking loss in [26] during the training step. At present, most supervised hashing methods train with pairs of samples to prove the similarities/dissimilarities of video pairs. These methods design loss functions to preserve pairwise similarity of videos. But some recent studies have shown that triplet-based similarities can get better results than pairwise similarities. In the training step we use the strategy in Section 3.3.1 to form a triple $\left(I,I^{+},I^{-}\right)$, I  is the query video, in the training set, $I^{+}$ and I are from the same category of video, $I^{-}$ and I are from different categories. By training we want to get a mappin*g* $F\left(\;\right)$, After mapping features to hamming space, the distance between F(I) and $F(I^{+})$ is less than F(I) and ${\mathrm{FI}}^{-}$. Gradient descent is required during supervised learning and training, using distance metrics such as hamming distance may cause the loss function to be nondifferentiable. For ease of optimization, we use Euclidian distance as distance metric in training step. In the retrieval phase, the binary codes of the videos are obtained, Hamming distance is used to measure the similarity between the videos due to the fast bit XOR operation. Finally, we define the triplet loss by Equation (3):

(3) $$L=\max\left\{0,∥F_{i}^{0}-F_{i}^{+}∥{}_{2}^{2}-F_{i}^{0}-F_{i}^{-}∥{}_{2}^{2}+\alpha \right\}$$

where $F_{i}^{0}$, $F_{i}^{+}$ and $F_{i}^{-}$ represent the feature vectors of the query video, similar video and dissimilar video, respectively. α is a margin parameter whose purpose is to ensure that there is enough divergence between the query-positive and query-negative. In our experiment, it is uniformly set to 1/4 of the hash code length. We use batch gradient decent to optimize the objective function in Equation (4):

(4) $$\underset{\theta }{\min}\overset{m}{\underset{i=1}{\sum }}L_{\theta }+{\lambda ∥\theta ∥}_{2}^{2}$$

where θ is the parameter to be solved, $L_{\theta }$ is the triplet loss, m is the quantity of samples in a mini batch, λ is a regularization parameter used to avoid overfitting.

#### 3.3.1. Triplet Selection

For a query video Q, we first select the videos {P_1_, P_2_, …, Pn} that belongs to the same class and shears the same label as it in the dataset in a mini-batch. This creates a set of query-positive video pairs (Q, P_1_), (Q, P_2_), …, (Q, Pn). The rest of the videos {V_1_, V_2_, …, Vn} in the mini-batch are not similar to the query video. Finally, we can form a series of triples consisting of a query-positive video pair and a dissimilar video like (Q, P_1_, V_1_), (Q, P_1_, V_2_), …, (Q, Pn, Vn), etc.

## 4. Experiments

In this section, we conduct experiments on three public video datasets to prove the effectiveness of our method. We start with introducing the datasets and the pre-trained network then show our experimental results with comparison to some representative hashing methods on video retrieval task.

### 4.1. Datasets and Pre-Trained Model

UCF-101 Dataset [54]

This consists of 101 categories realistic videos collected from YouTube. UCF-101 gives the largest diversity of classes among video datasets, the videos are divided into 25 groups, each consisting of 4–7 videos. Groups of videos have some common features, such as similar backgrounds, similar perspectives, and so on. The clip duration for most videos in UCF101 is less than 10 s. The training set of the original dataset is used for supervised learning and test set is used for retrieve.

JHMDB Dataset [55]

This database contains a total of 928 videos grouped into 21 categories. Most videos involve single actions such as throwing, walking and kicking a ball, and each category has 36–55 samples containing 15–40 frames. We follow the setting in [27], 10 videos each category are selected for training, 10 videos each category are selected for query, 20 videos each category are selected as gallery sets.

HMDB-51 Dataset [56]

It consists of a total of 7000 videos in 51 categories collected from YouTube, and each category contains at least 100 samples. The video samples come from a wide range of sources, most of which are from movie clips. The training sets are used for supervised learning and test sets for retrieve.

Pretrained model

We use the pre-trained 3D ResNet model [49] on the Kinetics dataset, which contains 300,000 videos to extract features.

### 4.2. Experimental Settings and Evaluation Metrics

#### 4.2.1. Experimental Settings

Three datasets are used to measure the performance of our method, split of the datasets is shown in Table 2. According to previous research experience [35,36,52], we use the method in Section 3.1.1 to select n non-overlapped video frames from each video. Then all the video frames are cropped to h×w. Therefore, the final input dimensions are c×n×h×w, where c is the channel number. In all of our experiment, we consistent set n=16, h=w=112. All experiments are performed on a workstation equipped with an Intel Xeon E5-2630 v3 CPU, 32 GB RAM and an NVIDIA Tesla K40c GPU. During the training phase, we used the SGD optimizer for gradient descent, the initial learning rate is 10^−4^, and the learning rate was reduced to 1/10 of the original rate every 40 epochs. The training epochs of UCF-101, JHMDB, HMDB are 150, 100, 150 the batch size of UCF-101, JHMDB, HMDB are set to 80, 60, 80.

#### 4.2.2. Evaluation Metrics

Mean Average Precision (mAP)

We employ mean average precision (mAP) to evaluate the performance of the proposed video retrieval algorithm, where average precision (*AP*) is calculated by Equation (5):

(5) $$AP=\frac{1}{R}\overset{n}{\underset{k=1}{\sum }}\frac{k}{R_{k}}\times rel_{k}$$

where n is the total number of the dataset, R represent the amount of videos that have relations to the retrieval videos in the dataset, and $R_{k}$ represent the quantity of the similar videos in the top k returns. When the video at position k is similar to the query $rel_{k}=1$; otherwise $rel_{k}=0$. Finally, mAP is obtained by calculate the mean value of *AP*.

Precision@N

This represents the proportion of correct retrieval results in the top-*N* retrieval results. The definition of precision@N is shown in Equation (6):

(6) $$Precision@N=\frac{\sum_{i=1}^{N}rel_{k}}{N}$$

where *N* is number of the top-*N* retrieved results, if the retrieved video similar to the query video $rel_{k}=1$; otherwise $rel_{k}=0$.

### 4.3. Experimental Results

To verify the performance of our algorithm, several state-of-the-art video retrieval methods are used to compare, including DVH [57], ITQ [42], DCNNH [3], SPDTH [27], BIDLSTM [28], DBH [22], DNNH [23].

#### 4.3.1. Experimental Results on UCF-101

The experimental results and the comparison with other hashing-based methods on UCF-101 dataset are shown in Table 3a,b. We can see a huge improvement when compared with the traditional hashing method ITQ. DVH, DCNNH, SPDTH are all deep learning-based hashing methods, where DVH obtains frame-wise CNN feature representation by passing a set of video frames to the convolutional and pooling layers, and then temporal fusion is conducted in the fully connected layers. The selection of frames may seriously affect the result. Compared with DVH the mAP and precision of our method increase by 7.7–9.3% and 2.8–4.7%. DCNNH uses CNN to do feature extraction from video frames, and then fushes them into video features by average weighting, thus completely ignoring the temporal information. Compared with the referenced DCNNH use 2D CNN to extract features, the mAP increased by 2.8–4.7%.

SPDTH uses CNN + RNN to extract features and generate hash codes through temporal-aware hashing, so that temporal information can be well preserved, but it is not easy to train due to too many parameters. Compared with SPDTH, our method still achieves an increase by 4.4–5.7% in mAP and 1.7–3.0% in precision with a simpler loss function.

In order to explore the relationship between the network depth and the retrieval accuracy, we also use 3D ResNet-18 under the UCF-101 dataset for the experiment, and the results are shown in Table 3c. It can be clearly seen from the table that a deeper network achieves better results. For each length of hash codes, the value of mAP increases by about 5%.

In order to demonstrate the effect of the hash layer, we remove the activation function in the hash layer, and directly use the full connection structure to map the video features into 64, 128 and 256 dimensions, and then compare the result with the previous one with a hash layer as shown in Table 3d. The results show that the value of mAP decreases obviously when the hash layer is removed, which proves the effectiveness of the hash layer.

#### 4.3.2. Experimental Results on JHMDB

The experimental results and the comparison with other hashing-based methods on JHMDB dataset are shown in Table 4a,b. The results on JHMDB are similar to those on UCF101, and our method still has a significant advantage over ITQ and DVH, even compared with the latest method BIDLSTM we also have a slight advantage about 0.3–2% in mAP. BIDLSTM also use a CNN + RNN model to extract video features, then map the features into binary space to get compact binary codes. A CNN model with stack heterogeneous convolutional multi-kernel is used to do feature extraction of the frames, then bidirectional long short term memory (LSTM) network is applied for maintaining the temporal information. Compared with BIDLSTM, the method we proposed is simpler in structure but more efficient for retrieval.

We also use different pre-trained networks on the JHMDB dataset to conduct experiments, and the results are shown in Table 4c. It is obvious from the results that increasing the depth of the network is helpful for retrieval.

The influence of the hash layer is also listed in Table 4d. The results obtained are similar to those on the UCF101 dataset, and the value of mAP decreases obviously after removing the hash layer. The results on the two datasets prove the effectiveness of the hash layer.

#### 4.3.3. Experimental Results on HMDB-51

The experimental results and the comparison with other hashing-based methods on HMDB-51 dataset are shown in Table 5a. Where, DBH and DNNH use the ResNet50 model for feature extraction of video frames, which is essentially a frame-based method. Compared with them, our method has obvious advantages due to the use of video-based features. The mAP of our method increases at least 6.2% compared with DNNH at 128 bits, and increases by 18.9% at most compared with DBH at 256 bits.

In order to prove the robustness of our method, we test the precisions of top-10, top-50 and top-100 respectively, the results are shown in Table 5b. It can be seen from Table 5b that when N increases from 10 to 50, the precision basically does not decline, but when N increases to 100, the precision begins to decline. This indicates that our method has stable precision for the videos in the front of the retrieval results.

In order to verify the influence of the number of video frames *n* on the performance of our method, we test the mAP when *n* equal to 12,16,20 respectively, the results are shown in Table 5c. The results show that when we extract 20 frames of each video, the mAP does not increase, but decreases. Increasing the sampling frequency of video frames does not necessarily improve the retrieval accuracy.

All the results prove that DSVH outperform many advanced video retrieval methods not only in accuracy but also in stability By selecting video frames and using 3D CNN, we can directly obtain the spatial-temporal feature in videos, and then get compact binary codes of the whole video under the supervised training, without considering how to maintain the temporal information after the convolution operation like other frame-based video retrieval methods, the use of pre-trained model also saves a lot of work and time.

## 5. Conclusions

In this paper, we have proposed an efficient video retrieval method named DSVH, which combines 3D convolution and supervised hashing for efficient video retrieval. By using 3D convolution for feature extraction from a series of video frames arranged in chronological order, and then using the supervised hashing method to map video features into Hamming space for similarity comparison, the retrieval performance is greatly improved. Compared with traditional methods such as ITQ [42], the mAP of our method increases at least 13.9% on HMDB-51, and at most 50.4% on UCF-101. Moreover, compared with the representative deep learning-based method SPDTH [27], the mAP of our method increases by 4.4–5.7%; 0.3–2% on UCF and JHMDB, and precision@N increases by 1.7–3%; 2.2–3.1% on UCF and JHMDB, respectively. A series of experimental results prove that our algorithm has superiority over many state-of-the-art video retrieval methods. For long video retrieval, we consider using more frames to extract features for each video in the future to achieve satisfactory results.

## Figures and Tables

**Table 1:** The details of the network architecture.

Layer Name	Architecture
Conv1 7×7×7,64, stride 1(T), 2(XY) 3×3×3 max pool, stride 2 Conv2_x $\left[\begin{array}{ll} 3\times 3\times 3, & 64\\ 3\times 3\times 3, & 64 \end{array}\right]\times 3$ Conv3_x $\left[\begin{array}{ll} 3\times 3\times 3, & 64\\ 3\times 3\times 3, & 64 \end{array}\right]\times 4$ Conv4_x $\left[\begin{array}{ll} 3\times 3\times 3, & 64\\ 3\times 3\times 3, & 64 \end{array}\right]\times 6$ Conv5_x $\left[\begin{array}{ll} 3\times 3\times 3, & 64\\ 3\times 3\times 3, & 64 \end{array}\right]\times 3$ Average pool, 400-d fc, softmax	

**Table 2:** Split of datasets.

Datasets	Training Set	Test Set	Gallery Set
UCF-101	9537	3783	-
JHMDB	210	210	410
HMDB-51	3570	1530	-

**Table 3:** Experimental results on UCF-101 dataset.

(a) Comparison Results of mAP on UCF-101 Dataset			
**Methods**		**UCF-101 Dataset**	
**Bits**	**64 Bits**	**128 Bits**	**256 Bits**
ITQ	0.294	0.305	0.306
DVH	0.705	0.712	0.729
DCNNH	0.747	0.783	0.796
SPDTH	0.741	0.750	0.762
DSVH (ours)	**0.798**	**0.801**	**0.806**
**(b) Comparison Results of Precision@N on UCF-101 Dataset**			
**Methods**		**Precision@30**	
**Bits**	**64 Bits**	**128 Bits**	**256 Bits**
ITQ	0.389	0.419	0.432
DVH	0.734	0.744	0.756
DCNNH	-	-	-
SPDTH	0.751	0.757	0.765
DSVH (ours)	**0.781**	**0.774**	**0.784**
**(c) Comparison Results of mAP with Different Network Depths**			
**Networks**		**UCF-101 Dataset**	
**Bits**	**64 Bits**	**128 Bits**	**256 Bits**
3D ResNet-18	0.735	0.756	0.757
3D ResNet-34	**0.798**	**0.801**	**0.806**
**(d) Influence of Hash Layer on mAP**			
**Networks**		**UCF-101 Dataset**	
**Bits**	**64 Bits**	**128 Bits**	**256 Bits**
With Hash Layer	**0.798**	**0.801**	**0.806**
Without Hash Layer	0.752	0.787	0.785

**Table 4:** Experimental results on JHMDB dataset.

**(a) Comparison Results of mAP on JHMDB Dataset**			
**Methods**		**JHMDB dataset**	
**Bits**	**16 Bits**	**32 Bits**	**64 Bits**
DVH	0.332	0.368	0.369
ITQ	0.132	0.145	0.151
SPDTH	0.386	0.438	0.464
BIDLSTM	0.410	0.455	0.482
DSVH (ours)	**0.430**	**0.458**	**0.494**
**(b) Comparison Results of Precision@N on JHMDB Dataset**			
**Methods**		**Precision@20**	
**Bits**	**16 Bits**	**32 Bits**	**64 Bits**
DVH	0.342	0.368	0.382
ITQ	0.152	0.175	0.191
SPDTH	0.375	0.408	0.438
BIDLSTM	-	-	-
DSVH (ours)	**0.397**	**0.430**	**0.469**
**(c) Comparison Results of mAP with Different Network Depths**			
**Networks**		**JHMDB Dataset**	
**Bits**	**16 Bits**	**32 Bits**	**64 Bits**
3D ResNet-18	0.371	0.411	0.444
3D ResNet-34	**0.397**	**0.430**	**0.469**
**(d) Influence of Hash Layer on mAP**			
**Networks**		**JHMDB Dataset**	
**Bits**	**16 bits**	**32 bits**	**64 bits**
With Hash Layer	**0.397**	**0.430**	**0.469**
Without Hash Layer	0.320	0.384	0.416

**Table 5:** Experimental results on HMDB-51 dataset.

**(a) Comparison Results of mAP on HMDB-51 Dataset**			
**Methods**		**HMDB-51 Dataset**	
**Bits**	**64 Bits**	**128 Bits**	**256 Bits**
ITQ	0.408	0.416	0.436
DBH	0.389	0.391	0.386
DNNH	0.487	0.503	0.493
DCNNH	0.458	0.451	0.467
DSVH (ours)	**0.562**	**0.565**	**0.575**
**(b) Comparison of Different N on Precision@ Based HMDB-51 Dataset**			
**Dateset**		**HMDB-51 Dataset**	
**Bits**	**64 Bits**	**128 Bits**	**256 Bits**
Precision@10	0.522	0.531	0.537
Precision@50	0.513	0.519	0.530
Precision@100	0.405	0.408	0.413
**(c) Comparison mAP of Different Frames on HMDB-51 Dataset**			
**Dateset**		**HMDB-51 Dataset**	
**Bits**	**64 Bits**	**128 Bits**	**256 Bits**
12 frames	0.490	0.516	0.539
16 frames	**0.562**	**0.565**	**0.575**
20 frames	0.527	0.550	0.556

## Data Availability

Data sharing not applicable.
